# Influence of intraoral scan extension on the accuracy of impressions with 3d-printed identical single tooth preparations

**DOI:** 10.1007/s00784-025-06645-2

**Published:** 2025-11-11

**Authors:** Andreas Magnus Geyer, Lara Anabell Gutjahr, Pablo Cores Ziskoven, James Deschner

**Affiliations:** Department of Periodontology and Operative Dentistry, University Medical Center of the Johannes Gutenberg-University Mainz, Augustusplatz 2, Mainz, 55131 Germany

**Keywords:** Intraoral scan, Digital dentistry, Preparation, 3d print

## Abstract

**Objectives:**

This study investigated whether the accuracy of the digital impression of identical single-tooth preparations is influenced by the size of an intraoral scan.

**Materials and methods:**

The intraoral scans of 30 subjects with identical preparations for a molar and a premolar were digitally processed, 3d-printed and rescanned in different scan extensions. The scans of the entire dental arch, the half arch and the sequence from the preparation with adjacent teeth were examined. The approximal and occlusal distances between the preparation and the adjacent teeth were measured, as were the volumes of the prepared teeth. For statistical analysis, A Friedman test was performed for the precision measurements and a Wilcoxon signed-rank test for the trueness measurements.

**Results:**

The original planning differed significantly from the measurements of the volumes (*p* < 0.001) and the occlusal distances of the molar and premolar (*p* ≤ 0.003). The sequence scan differed significantly when comparing the volumes of the molar (*p* < 0.001), the approximal distance measurement of the premolar (*p* < 0.001) and the occlusal distance measurement of the premolar (*p* < 0.001). The measurements of the half arch had the smallest median deviations (approximal ≤ 26 μm, occlusal ≤ 102 μm) from the original planning, compared to the sequence (approximal ≤ 26 μm, occlusal ≤ 131 μm) and full arch scan (approximal ≤ 27 μm, occlusal ≤ 122 μm).

**Conclusions:**

Small scan extensions can provide more accurate results than larger scan sizes, whereby approximal distances are recorded more precisely than occlusal ones.

**Clinical relevance:**

3d-printing and intraoral scanning with small scan sizes can reproducibly achieve clinically acceptable accuracies with high consistency.

## Introduction

Digital impressions using intraoral scanners (IOS) are becoming an increasingly popular alternative to conventional impressions. The IOS is generally faster to obtain than a conventional impression, is reproducible as a digital model and can be carried out with interruptions [1]. It can also be used as a support during preparation, be it through the scalability of the image for better visualization or through algorithms for detecting undercuts and preparation angles [2]. In addition, digital impressions are often perceived as more comfortable by patients [1, 3].

Modern IOS are comparable to conventional impressions in terms of accuracy, especially if sufficient hard tissue is available as a reference for superimposing the images [4, 5]. Probably the most common application for intraoral scanning is the prosthetic restoration of lost tooth substance. A highly precise impression is an important prerequisite for the functional and aesthetic restoration of teeth.

The IOS as a composition of light images can be influenced by numerous optically specific phenomena such as reflection, optical transmission, scattering or absorption [6]. To obtain precise information about the dimensions of the objects being scanned, modern intraoral scanners use confocal imaging techniques. This involves the use of a light source and a detector that captures reflected light through a focal point. The position and size of the focal point allow the light rays to be filtered so that only light from a specific depth, the focal plane, is captured by the detector. The filtered images can then be analysed to determine features or points of interest such as defined contrasts or distinctive structures. Matching features can then be used to sort multiple images according to their position. This generates a point cloud, which is supplemented by surfaces and textures on the software side to display a 3 d object. The density of the point cloud and the resulting size of the triangulated surfaces are directly related to the resolution that a scanner can display. However, it does not provide any information about the accuracy of the system used [7].

The accuracy of the digital mesh is influenced by the process of translating 2 d images into 3 d objects using scanner-specific software. Depending on the size of the object, inaccuracies can add up and affect the overall result [8].

An exact scan is an important prerequisite for all subsequent work, be it digital control and planning or as a basis for models in the conventional fabrication of dentures or restorations [9].

Although studies already indicate that the accuracy of the scan size depends on the number of missing teeth per jaw, to our knowledge it has not yet been investigated whether the different scan extensions can influence the acquisition of clinically important parameters such as the approximal and occlusal distance to the surrounding tissue when healthy teeth are present [10, 11]. This study investigated whether the accuracy of the digital impression with identical single-tooth preparations is influenced by the size of an intraoral scan. Therefore, the null hypothesis of the following study is that scan size has no statistically significant influence on scan accuracy for single tooth preparations when capturing occlusal and approximal distances.

## Materials and methods

This study was approved by the Ethics Commission University Medical Center of the Johannes Gutenberg University Mainz (Registration No.: 2025–18007). The informed consent was obtained from each participant prior to enrolment in the study.

To produce the models, digital impressions of the upper and lower jaw were taken from a total of 30 healthy subjects using an intraoral scanner (Primescan, Dentsply Sirona, USA). The scanner was calibrated according to the manufacturer’s standards prior to each use. Each scan begins by focusing on the oral surface at a 45-degree angle to the tooth axis on a terminal molar. After scanning the oral surfaces, the scanner was rotated to capture the occlusal surface, scanning back towards the terminal molar to ensure complete coverage. Next, the scanner was rotated towards the vestibular surfaces and moved back to where the oral scan had been completed. Finally, any missing data is filled in to ensure the scan is coherent. The occlusion was recorded by scanning the vestibular surface of the teeth in a wave-like pattern with a firm bite.

A molar (tooth 16) and a premolar (tooth 24), according to the notation of the Federation Dentaire Internationale (FDI), were digitally replaced by idealized preparations with approximal and occlusal markings for later evaluation. The markings are cylindrical elevations with a diameter of 0.5 mm to enable consistent measurement of the occlusal and approximal distances between the different models. The subjects had no approximal or occlusal restorations on the surrounding teeth. These scans were provided with supports to stabilize the recorded bite after printing using a CAD/CAM software for dental applications (Exocad 3.2 Elefsina, Exocad GmbH, Germany).

**Fig. 1:**
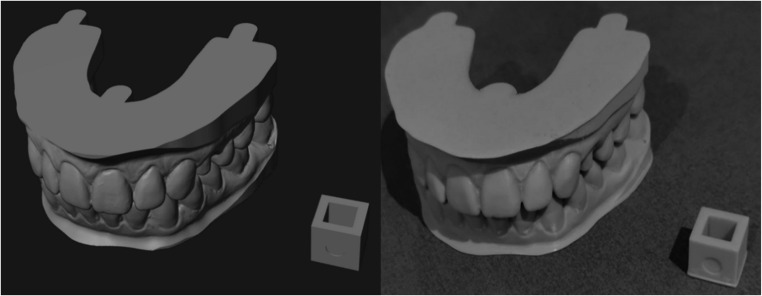
Model of a proband with a cube during planning and after 3d-printing

After calibration, the models were printed using dental model resin (KeyModel Ultra Light Grey, Keystone Industries, USA), according to the manufacturer’s instructions, on a high-resolution 3 d printer (Phrozen Sonic Mini 8 K S, Phrozen Tech Co., Taiwan). The consistency of the print resolution was checked with 10 × 10 × 10 mm cubes, which were attached to each print batch and then measured with a micrometer in the X, Y and Z-axis (Fig. 1).

**Fig. 2:**
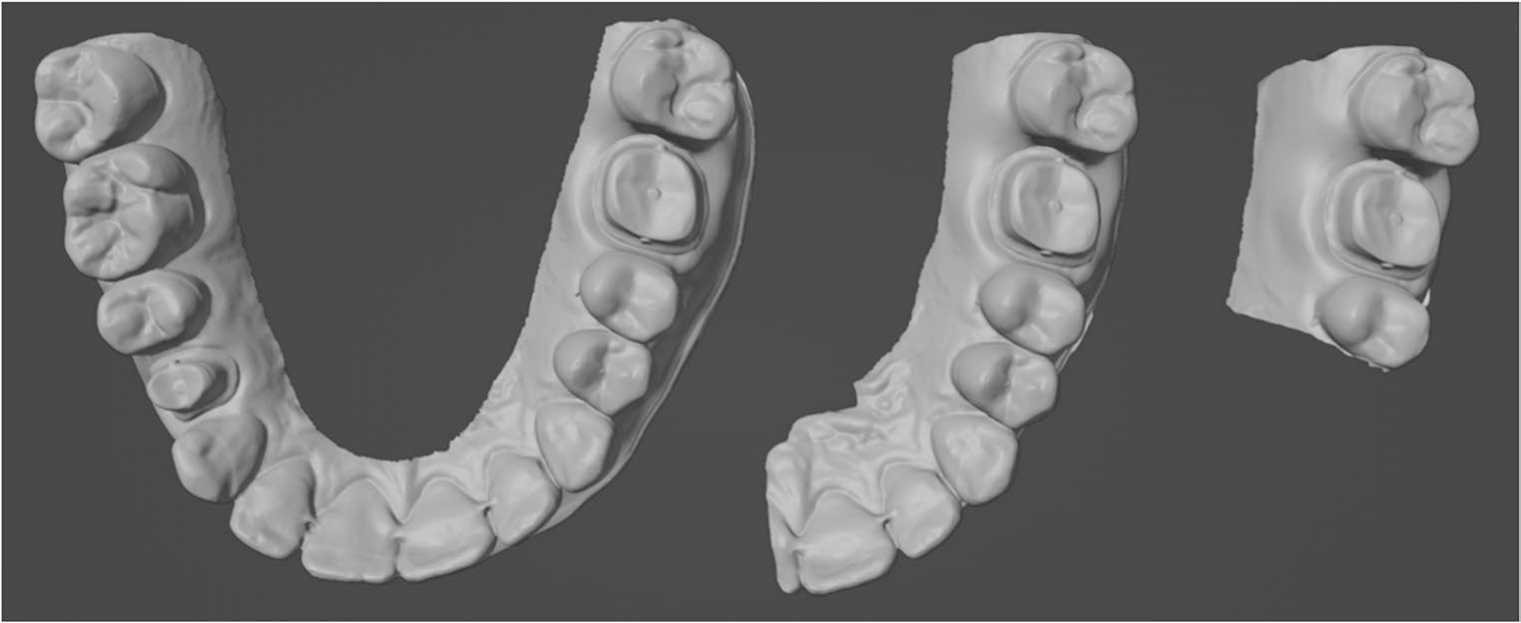
Intraoral scan as full arch, half arch and sequence of a proband in the upper jaw

After the print, the prepared models were scanned again with the same intraoral scanner and scan strategy in three different sizes: the full dental arch (FA) in the upper and lower jaw, half of the dental arch (HA) and finally the sequence (S) with the preparations of the molar and premolar and the respective adjacent teeth (Fig. 2). The opposite bite was scanned in the same extension. The resolution of the scanner was analysed by measuring the median of the triangle sizes, the median of the distances between vertices, and the smallest distance between two points.

The models were then superimposed on the identical preparations of the molar and the premolar using an iterative closest point algorithm with a 3d-modeling software (Blender, Blender Foundations, Netherlands). The superimposition was performed to slice all scans with different extensions at the identical position for a volume analysis of the preparations. The volume analysis was performed to verify the reproducibility of the scans in the same software. The identical preparations were cut out at their preparation margins and the volume of the resulting sections were compared with each other. The measured volumes of the 30 probands were divided into two separate groups for the molar (VolM) and the premolar (VolP).

**Fig. 3:**
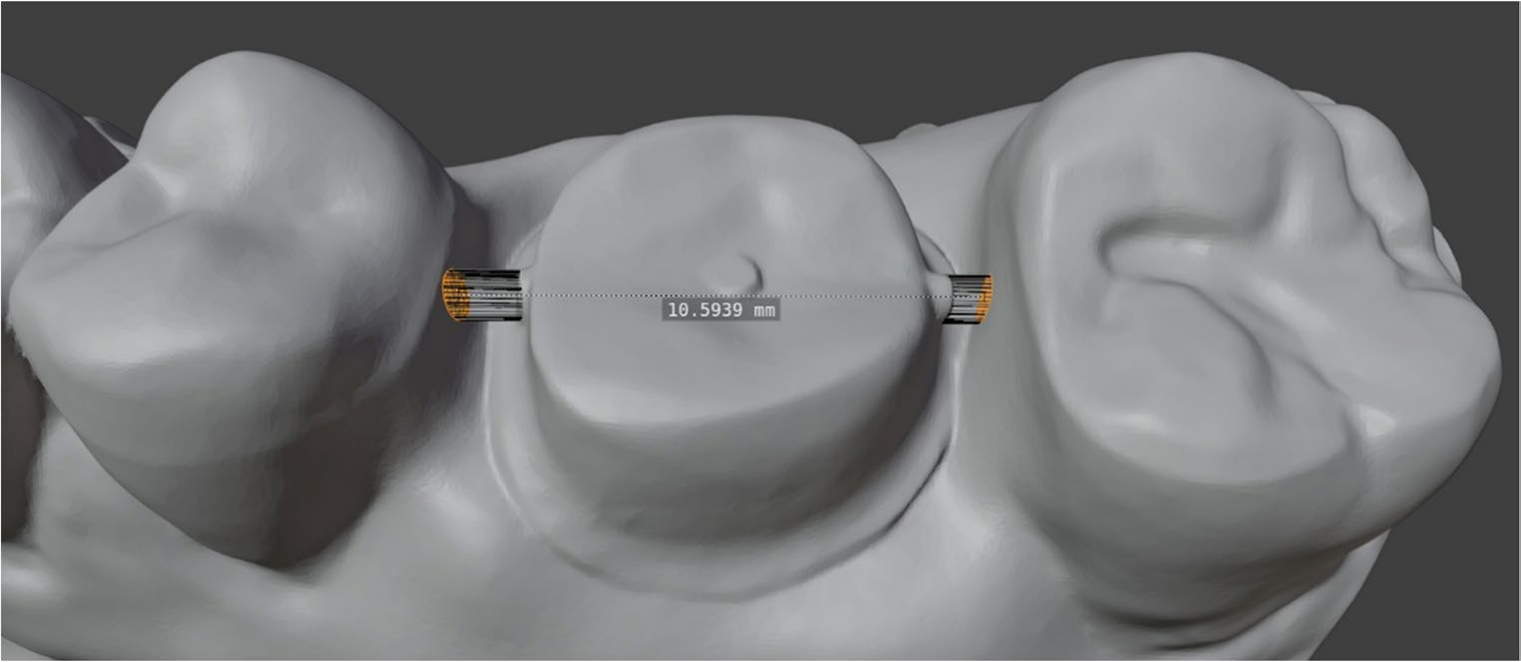
Approximal distance measurement with a 0.5 diameter surface selection

The distance between the adjacent teeth and the occlusal distance were measured from the marked surfaces by averaging the distance of all recorded data points on the adjacent teeth within a circular area with a diameter of 0.5 mm to compensate for possible artifacts in the scans (Fig. 3). The recorded data points were the intersection of the elongation of the approximal and occlusal markings extended to the surface of the surrounding teeth.

The measurements of the approximal and occlusal distances of the molar and premolar were divided into four groups. The first group examined the approximal distances at the molar (AppM), the second the approximal distances at the premolar (AppP), the third the occlusal distances at the molar (OccM) and the fourth the occlusal distances at the premolar (OccP). Each of these groups contained the 30 measured values of the different probands for FA, HA and S. The measurements were compared between the groups for the evaluation of precision and in relation to the original planning for the evaluation of trueness.

The data were used to check for statistically significant differences in the scanning methods. A Wilcoxon signed-rank test was performed on each of the FA, HA and S scans, comparing them with the corresponding planned files for occlusal, approximal and volumetric measurements.

A repeated measures analysis using the Friedman test was performed to determine the effect of scanning and 3d-printing on precision. Following the Friedman test, pairwise comparisons were conducted using the Bonferroni correction to examine the results for significant differences.

## Results

The 10 × 10 × 10 mm cubes which were printed to check the resolution consistency returned 10.000 ± 0.014 mm in the X-axis, 10.000 ± 0.012 mm in the Y-axis and 9.061 ± 0.079 mm in the Z-axis. The deviation in height resulted in a 9.39% decrease after printing.

The median triangle size for all scans was 5.23 µm^2^ with an interquartile range (IQR) of [5.08; 5.51]. The median distance between two measured vertices was 120 μm with the IQR [118; 123]. The smallest distance between two vertices was measured for every scan. The median value for all scans combined was 6 μm with the IQR [4; 10]. The distances between vertices varied across the scans. In areas with prominent features such as edges and creases, the triangle size was smallest and the point density was greatest. In contrast, on flat surfaces, the point density was lowest.

**Fig. 4:**
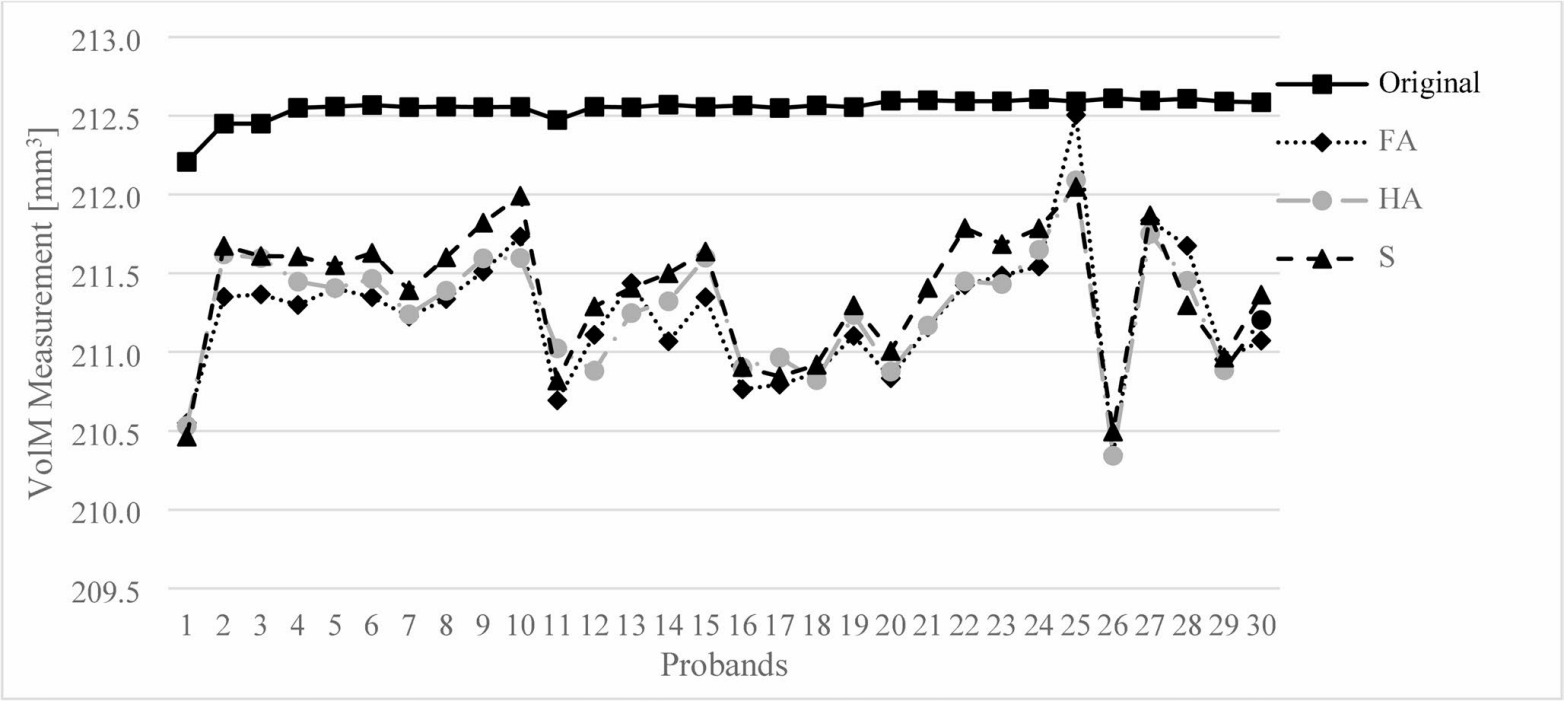
Measurement of VolM for the 30 probands compared to the original planning

The measured volumes of VolM all differed significantly from the original planning (*p* < 0.001) (Fig. 4). The S scan showed significant differences to the FA scan (*p* < 0.001) and the HA scan (*p* < 0.001). The volumes measured after printing and scanning differed from the planned model by 1.237 mm³ (median) and IQR [1.102; 1.607] in the FA, by 1.209 mm³ (median) and IQR [0.996; 1.551] in the HA, and by 1.109 mm³ (median) and IQR [0.858; 1.521] in the S.

**Fig. 5:**
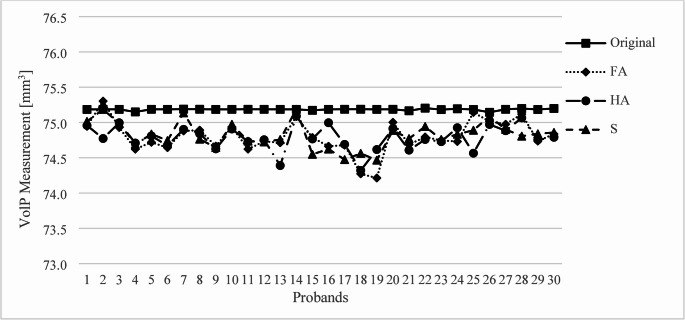
Measurement of VolP for the 30 probands compared to the original planning

The measured volumes of VolP also all differed significantly from the original planning (*p* < 0.001) (Fig. 5). There were no significant differences between the FA, HA and S scan. The volumes of VolP measured after printing and scanning differed from the planned model by 0.423 mm³ (median) and IQR [0.242; 0.501] in the FA, by 0.411 mm³ (median) and IQR [0.274; 0.488] in the HA, and by 0.376 mm³ (median) and IQR [0.267; 0.458] in the S.

There were no significant differences in the AppM measurements between the scanning methods and the original planning. In the AppM measurement of the approximal contacts, the scans deviated from the planned models by 0.027 mm (median) and IQR [0.018; 0.061] in the FA, by 0.022 mm (median) and IQR [0.013; 0.047] in the HA, and by 0.026 mm (median) and IQR [0.014; 0.040] in the S.

The OccM measurements differed significantly from the original planning in the FA (*p* < 0.001), HA (*p* < 0.001) and S (*p* < 0.001). The scans did not differ significantly from each other in their measurements. The occlusal contacts of the FA scan differed by 0.122 mm (median) and IQR [0.067; 0.208] from the planning, the HA by 0.102 mm (median) and IQR [0.064; 0.184], and the S by 0.131 mm (median) and IQR [0.065; 0.182].

The AppP measurement showed a significant difference between FA and S (*p* < 0.001). There were no significant differences in the FA-HA or HA-S comparisons, nor in those with the original planning. The approximal contacts deviated from the planning by 0.020 mm (median) and IQR [0.011; 0.042] in the FA scan; by 0.026 mm (median) and IQR [0.012; 0.038] in the HA and by 0.020 mm (median) and IQR [0.008; 0.026] in the S.

In the OccP measurement, the FA scan differed significantly from the planning (*p* = 0.003), as well as from the HA (*p* = 0.002) and S (*p* < 0.001) scans. However, the comparison of the planning to HA and S did not show any significant differences. HA and S also did not differ significantly from each other. In the occlusal measurement, the FA scan differed by 0.119 mm (median) and IQR [0.054; 0.169] from the original planning, as did the HA scan by 0.090 mm (median) and IQR [0.033; 0.160], and the S scan by 0.091 mm (median) and IQR [0.033; 0.160].

The volumes differed from each other by an average of 0.167 ± 0.108 mm^3^ at the molar and 0.123 ± 0.121 mm^3^ at the premolar.

On average, the deviation in the approximal measurements for all molars and premolars combined was 19 ± 15 μm between the different scan sizes. For the occlusal distances, the measurements of the different scan extensions differed by an average of 40 ± 27 μm. The measured values between the reference planning and the scanned models after 3d-printing were on average 33 ± 30 μm approximal and 128 ± 94 μm occlusal.

## Discussion

The study into whether the scan size has an influence on the accuracy of clinically important parameters such as approximal and occlusal contacts showed that smaller scan sizes are preferable for single-tooth preparations. The scan of the sequence was significantly more accurate than the full or half arch scan when analyzing the volume of the molar and measuring the occlusal and approximal distances of the premolar. The scan sizes of the half dental arch and the sequence were in general more accurate than the scan of the full dental arch. One possible explanation for this could be the number of captured images that are combined to create a 3 d object. When these images are combined, errors can add up, ultimately leading to measurable inaccuracies [12].

The clinically acceptable gap at the preparation margin for dental restorations is generally considered to be less than 120 μm [13]. Normal dental floss, which is approximately 30–50 μm wide, should fit under the approximal contact between the restoration and the adjacent tooth. The distances measured in this study, which represent the total mesial-to-distal approximal distance and are less than the width of a standard dental floss, demonstrate the accuracy of single jaw scans and thus confirm their clinical acceptance. However, the accuracy of the scans is reduced for occlusal distances, primarily due to the alignment of the upper and lower jaws, which is why subsequent occlusal correction of dentures is still necessary in some cases.

Using a preparation margin that is epigingival or slightly supragingival, as was done in this study, can have a negative effect on the scan. Supragingival margins are optimal for 3d-scanning as they are easier to capture accurately, being above the gum line and less affected by soft tissue interference. This leads to better visibility and consistency and reduces the potential for errors in the digital impression. In contrast, subgingival margins, which are closer to the gum line, can be more challenging to scan accurately due to interference from the surrounding tissue [14].

The presence of adjacent teeth can restrict intraoral scanners’ ability to capture the preparation from the optimal angles, which reduces accuracy. The scanner’s line of sight is obstructed, which makes it difficult to obtain precise 3 d data of the margins and surfaces of the preparation. This issue is particularly pronounced in deep preparations affecting the approximal surfaces [15]. The accuracy of intraoral scans can also be influenced by the imaging technology of the scanner used and the distance between the scanner and the surface to be scanned. Ideally, a scanning distance of 5–10 mm from the preparation is recommended, but this is not always possible due to varying heights of tooth preparations. The three most common technologies are: confocal microscopy, which offers high accuracy but longer scanning times; parallel confocal technology, offering a balance between accuracy and speed; and light triangulation, which is faster but in general less accurate than the other two methods [16, 17]. Depending on the application and scanner, the recording distance can therefore also have an impact on accuracy.

The measured approximal distances and the occlusal distances supported the values previously measured in other studies on the precision of the intraoral scanner used [18–20]. The trueness of the measured values was influenced by the 3d-print in addition to the scan sizes and was therefore measured in reference to this. The measurements of the approximal distances were significantly closer to the planning than the occlusal ones.

The measured occlusion could be influenced by the orientation of the models during printing, as the models were printed parallel to the occlusal plane. In the case of print layers printed with a layer thickness of 50 μm, printing artifacts may occur due to a lack of adhesion of the print layer. This usually affects the last layer, especially if it is a large area printed at the same time, which must adhere to the structures already printed. In addition, printing is limited by the resolution of the layer thicknesses, with 50 μm being the layer thickness recommended by the manufacturer. The resolution on the X and Y axes depends on the screen resolution. In this case, the screen resolution of the 3 d printer was 22 μm. It is generally recommended to choose a layer thickness of 50–100 μm for dental 3d-printing due to the higher conversion rate and stability [21, 22]. For 3d-printed models, the orientation of the planning should therefore be taken into account and the margin in the Z-axis should be extended accordingly.

Although all models were printed with the same layer thickness, these layers were not visible when scanned again. This may be because 3 d scanners have difficulty recognizing sharp edges due to their resolution, as well as due to errors in the software post-processing stage [23]. Although intraoral scanners are highly accurate, sharp edges are more difficult to capture precisely and must therefore also be taken into account during preparation [24].

The reference cubes were printed flat on the printing platform, which led to compression in the base layers. This influenced the final height of the cubes, which is why the measured Z-axis of the cubes deviated from the planning by approx. 1 mm. This has no influence on the tooth models, as after the first six bottom layers recommended by the manufacturer, the subsequent layers are consistent and hardly contribute to further distortions of the model. The bottom layers are important for the adhesion of the 3d-print to the platform and therefore have longer exposure times than the other layers.

**Fig. 6:**
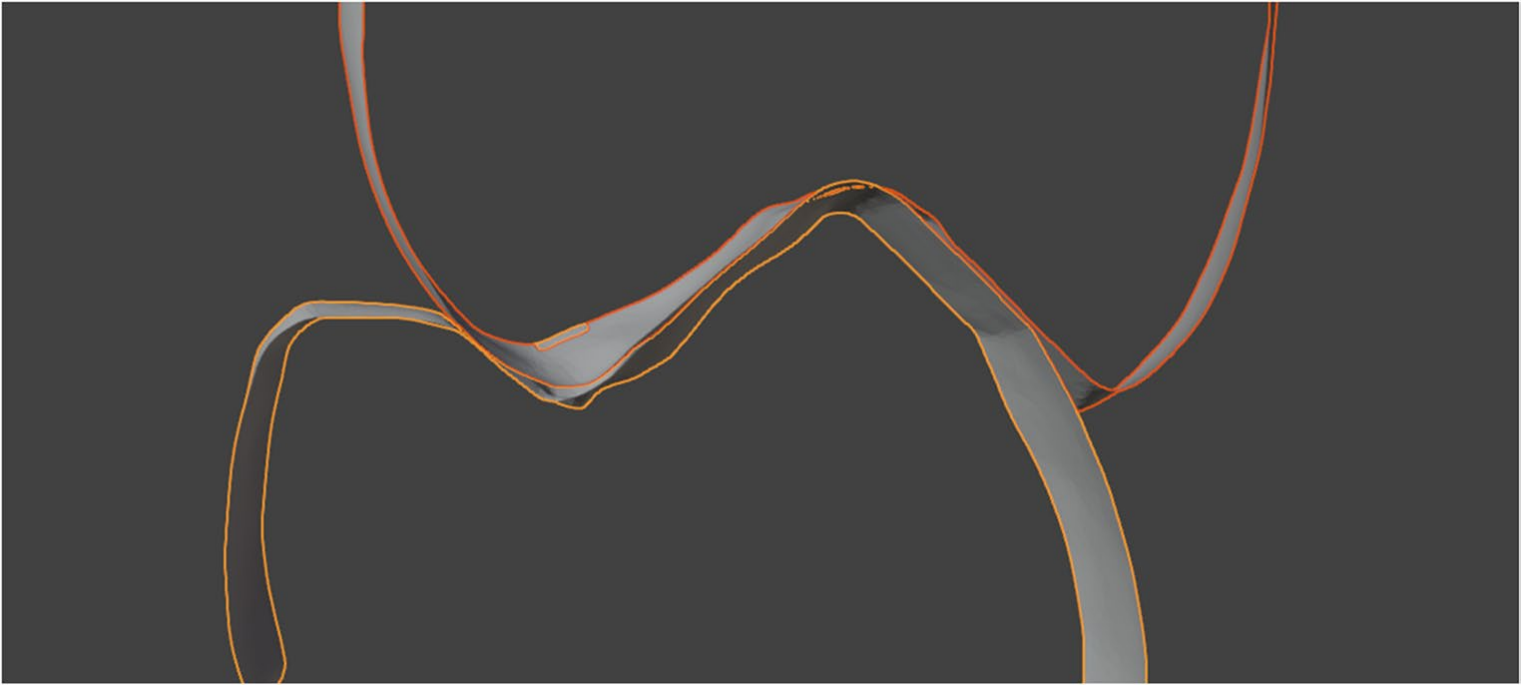
Penetration of the occlusal contact of two premolars in the reference scan

Another important factor that influences the accuracy of occlusal distances is how the scanner captures the alignment of the upper and lower jaw occlusion. The occlusal contacts captured by the intraoral scanner are defined as surface penetration after the upper and lower jaws have been aligned in the bite scan (Fig. 6).

This surface penetration is based on inaccuracies in the alignment of the upper and lower jaw when the vestibular surfaces are scanned. These penetrations are caused by the matching of the upper and lower jaw and are usually compensated for when creating dentures, but not when 3d-printing the models. These errors can substantially affect the occlusion, depending on the scanner used and the material of the models produced [25–27].

Given its inherent high trueness and precision, the choice of the Primescan could serve as a benchmark for high-accuracy studies. However, it also has the potential to create a skewed representation of overall scanner performance. In other studies, differences in scanner accuracy could lead to imbalanced conclusions, making the Primescan appear disproportionately favourable compared to other IOSs [28].

The volume analysis proved to be consistent in comparison between the models and between the original planning, where the print of the premolar showed a higher trueness and precision. When comparing the numerical deviations in percent, the volumes of the printed and scanned models deviated from the planning by 0.006% for the molar and 0.005% for the premolar. The observed consistent shrinkage of the models compared to the planning can be compensated for future printing processes by calibrating the printing parameters.

## Conclusion

This study showed that the accuracy of digital impressions of identical single-tooth preparations is influenced by the size of an intraoral scan. The accuracy of all different scan extensions was within a clinically acceptable range. The 3d-printing showed high consistency, but led to a minor shrinkage of the model, especially in the occlusal areas. Overall, the half-arch and sequence measurements were numerically more accurate than the full-arch scan. A small scan size with the surrounding tissue can therefore be recommended for single tooth preparations.

## Data Availability

No datasets were generated or analysed during the current study.
